# Intravitreal Adalimumab in Retinitis Pigmentosa: A Prospective Pilot Study Assessing Ocular Safety and Feasibility

**DOI:** 10.7759/cureus.109091

**Published:** 2026-05-18

**Authors:** Rubens C Siqueira, Tainara S Pinho, Cinara C Brandao

**Affiliations:** 1 Postgraduate Department, Faculdade de Medicina de São José do Rio Preto, Sao Jose do Rio Preto, BRA; 2 Immunogenetics Laboratory, Department of Dermatological, Infectious, and Parasitic Diseases, Faculdade de Medicina de São José do Rio Preto, Sao Jose do Rio Preto, BRA

**Keywords:** ellipsoid zone, erg, intravitreal adalimumab, oct, pilot study, retinitis pigmentosa, visual field

## Abstract

Objective

The objective of this study was to evaluate the short-term ocular safety, feasibility, and exploratory functional signals associated with intravitreal adalimumab (ADA) in patients with retinitis pigmentosa (RP).

Methods

This prospective, single-arm pilot study included 21 patients with RP who received intravitreal adalimumab (2 mg/0.05 mL) at baseline, month 2, and month 4, with follow-up to month 6. The primary objective was to assess ocular safety and treatment feasibility. Exploratory functional outcomes included best-corrected visual acuity (BCVA, logarithm of the minimum angle of resolution (LogMAR)) and automated perimetry parameters (mean deviation (MD), pattern standard deviation (PSD), and Field Preservation Deviation Index (FPDI)). Structural assessment was performed using optical coherence tomography (OCT). Analyses compared baseline and month 6 outcomes using paired statistical methods. BCVA values were analyzed at the patient level as the mean of both eyes.

Results

All scheduled intravitreal injections were successfully completed, and no serious ocular adverse events were observed during follow-up. Mean BCVA showed no statistically significant change from baseline to month 6 (0.76 to 0.70 LogMAR; p = 0.115). Visual field parameters (MD, PSD, and FPDI) remained stable, with no statistically significant differences over time. OCT findings demonstrated advanced baseline structural alterations consistent with RP, without evidence of treatment-related anatomical deterioration or cystoid macular edema during follow-up. Flicker electroretinography (ERG) responses were measurable in a limited subset of patients and were analyzed descriptively, showing no consistent evidence of decline.

Conclusions

In this prospective pilot study, intravitreal adalimumab demonstrated a favorable short-term ocular safety profile and was feasible to administer in patients with retinitis pigmentosa. No significant functional or structural changes were observed over six months. These findings are hypothesis-generating and support the need for controlled studies to further evaluate the safety and potential role of tumor necrosis factor alpha (TNF-α) inhibition in inherited retinal degeneration.

## Introduction

Retinitis pigmentosa (RP) comprises a heterogeneous group of inherited retinal dystrophies characterized by progressive degeneration of photoreceptors, typically beginning with rod dysfunction followed by secondary cone involvement. Clinically, this leads to nyctalopia, progressive constriction of the visual field, and, in advanced stages, central vision loss [1]. With an estimated prevalence of approximately 1 in 4,000 individuals, RP remains one of the leading causes of inherited blindness worldwide and represents a significant clinical and socioeconomic burden [2]. Despite advances in gene-specific therapies, including adeno-associated viral (AAV)-mediated gene augmentation, as well as emerging cell-based and prosthetic approaches, most patients still lack broadly applicable treatments capable of modifying disease progression across the diverse genetic spectrum of RP [1-3].

In addition to its genetic basis, RP is increasingly recognized as a condition associated with chronic para-inflammatory processes in the outer retina. Experimental and clinical studies have demonstrated microglial activation, increased expression of pro-inflammatory mediators, including tumor necrosis factor alpha (TNF-α), and disruption of the blood-retinal barrier, all of which may contribute to secondary photoreceptor degeneration [3-12]. This inflammatory microenvironment has been associated with oxidative stress, complement activation, and progressive cone loss following primary rod degeneration [7,9]. Within this framework, modulation of inflammatory pathways, including TNF-α signaling, may represent a biologically plausible target for investigation in inherited retinal degeneration.

Adalimumab (ADA) is a fully human monoclonal antibody directed against TNF-α and is widely used in systemic inflammatory diseases. In ophthalmology, both systemic and local administration of anti-TNF-α agents have demonstrated acceptable safety profiles in selected inflammatory conditions, including non-infectious uveitis [10,13-18]. Preclinical studies have also suggested that TNF-α inhibition may attenuate inflammatory cascades and photoreceptor cell death in experimental models of retinal degeneration [19-24]. However, clinical evidence regarding the intravitreal use of anti-TNF-α therapies in inherited retinal diseases remains limited, and their role in this context has not been established.

Given this biological rationale and the absence of widely effective mutation-independent therapies, exploratory clinical investigations are warranted. The present study was therefore designed as a prospective, single-arm pilot study to evaluate the ocular safety, feasibility, and exploratory functional outcomes associated with intravitreal adalimumab in patients with retinitis pigmentosa. Outcomes included best-corrected visual acuity (BCVA, logarithm of the minimum angle of resolution (LogMAR)), automated perimetry parameters (mean deviation (MD), pattern standard deviation (PSD), and Field Preservation Deviation Index (FPDI)), and optical coherence tomography (OCT) findings, with analyses focused on pre- to post-treatment changes over a six-month follow-up period. These results are intended to provide preliminary data to inform the design of future controlled clinical trials.

## Materials and methods

Study design and ethical approval

This was a prospective, single-arm, exploratory pilot study designed to evaluate the ocular safety, feasibility, and exploratory functional outcomes associated with intravitreal adalimumab in patients with retinitis pigmentosa (RP). The study was conducted in accordance with the tenets of the Declaration of Helsinki and Good Clinical Practice guidelines.

Ethical approval was obtained from the Brazilian National Research Ethics Committee (CONEP) (CAAE: 86943125.0.0000.0317), and the study was prospectively registered at ClinicalTrials.gov (Identifier: NCT07348588; Unique Protocol ID: ADARET). All participants provided written informed consent prior to inclusion.

Participants

Adult patients (≥18 years) with a clinical diagnosis of retinitis pigmentosa based on characteristic fundoscopic findings and multimodal imaging were eligible for inclusion. Inclusion criteria required measurable best-corrected visual acuity (BCVA) and reliable automated perimetry testing, as well as adequate media clarity to allow optical coherence tomography (OCT) acquisition.

Patients were excluded if they presented with active or recent intraocular inflammation or infection, choroidal neovascularization, uncontrolled glaucoma, known hypersensitivity to adalimumab, or any ocular or systemic condition that could interfere with study outcomes. Genotypic data were collected when available for exploratory purposes.

Intervention

All participants received intravitreal adalimumab (Humira®, AbbVie Inc., Chicago, USA) at a dose of 2 mg/0.05 mL administered at baseline (M0), month 2 (M2), and month 4 (M4). Injections were performed under sterile conditions using topical anesthesia and 5% povidone-iodine antisepsis. The injection site was located 3.5 mm posterior to the limbus in pseudophakic eyes and 4.0 mm in phakic eyes.

Outcome measures

The primary outcomes of the study were ocular safety and the feasibility of treatment administration. Safety endpoints included the occurrence of intraocular inflammation, endophthalmitis, retinal vasculitis, vitreous hemorrhage, cataract progression, and sustained intraocular pressure elevation.

Secondary exploratory outcomes included best-corrected visual acuity (BCVA, LogMAR), visual field parameters (mean deviation (MD), pattern standard deviation (PSD), and Field Preservation Deviation Index (FPDI)) and structural retinal assessment using OCT. Central macular thickness was quantitatively evaluated, while ellipsoid zone integrity was assessed qualitatively in cases where measurement was not feasible.

Flicker electroretinography (ERG, 30 Hz) was included as an exploratory outcome. Due to advanced disease stage, ERG responses were measurable only in a limited subset of patients and were therefore analyzed descriptively.

Assessment schedule

Patients were evaluated at baseline (M0), month 2 (M2), month 4 (M4), and month 6 (M6). Safety assessments were additionally performed 7 to 14 days after each intravitreal injection.

Visual field and imaging

Automated perimetry was performed using the iCare COMPASS system (Icare Finland Oy, Vantaa, Finland), with testing strategies selected according to disease severity (10-2 or 24-2 grids). Optical coherence tomography was performed using the same device throughout the study to ensure consistency. Segmentation errors were manually reviewed and corrected when necessary.

Statistical analysis

Statistical analyses were performed using R (version 4.3.2; R Foundation for Statistical Computing, Vienna, Austria) and IBM SPSS Statistics (version 29.0.1; IBM Corp., Armonk, USA). Normality of data distribution was assessed using the Shapiro-Wilk test. Paired comparisons between baseline and month 6 were conducted using either paired t-tests or Wilcoxon signed-rank tests, as appropriate.

Given the exploratory nature of the study and the limited sample size, statistical analyses were considered descriptive and hypothesis-generating. No adjustment for multiple comparisons was performed.

Data handling

All patient data were de-identified prior to analysis and stored in secure institutional systems with restricted access. No patient-identifiable information was included in figures or reports.

## Results

A total of 21 patients diagnosed with retinitis pigmentosa were included in the analysis after completing all scheduled visits and examinations.

Functional and structural outcomes were assessed through best-corrected visual acuity (BCVA) in LogMAR, automated perimetry parameters including mean deviation (MD), pattern standard deviation (PSD), and Field Preservation Deviation Index (FPDI), as well as 30 Hz flicker electroretinography (ERG) and optical coherence tomography (OCT).

These multimodal evaluations provided complementary measures of retinal function and morphology, enabling the assessment of central and peripheral vision, electrophysiological integrity, and retinal microstructure before and after intravitreal adalimumab treatment.

Visual acuity analysis

**Figure 1:**
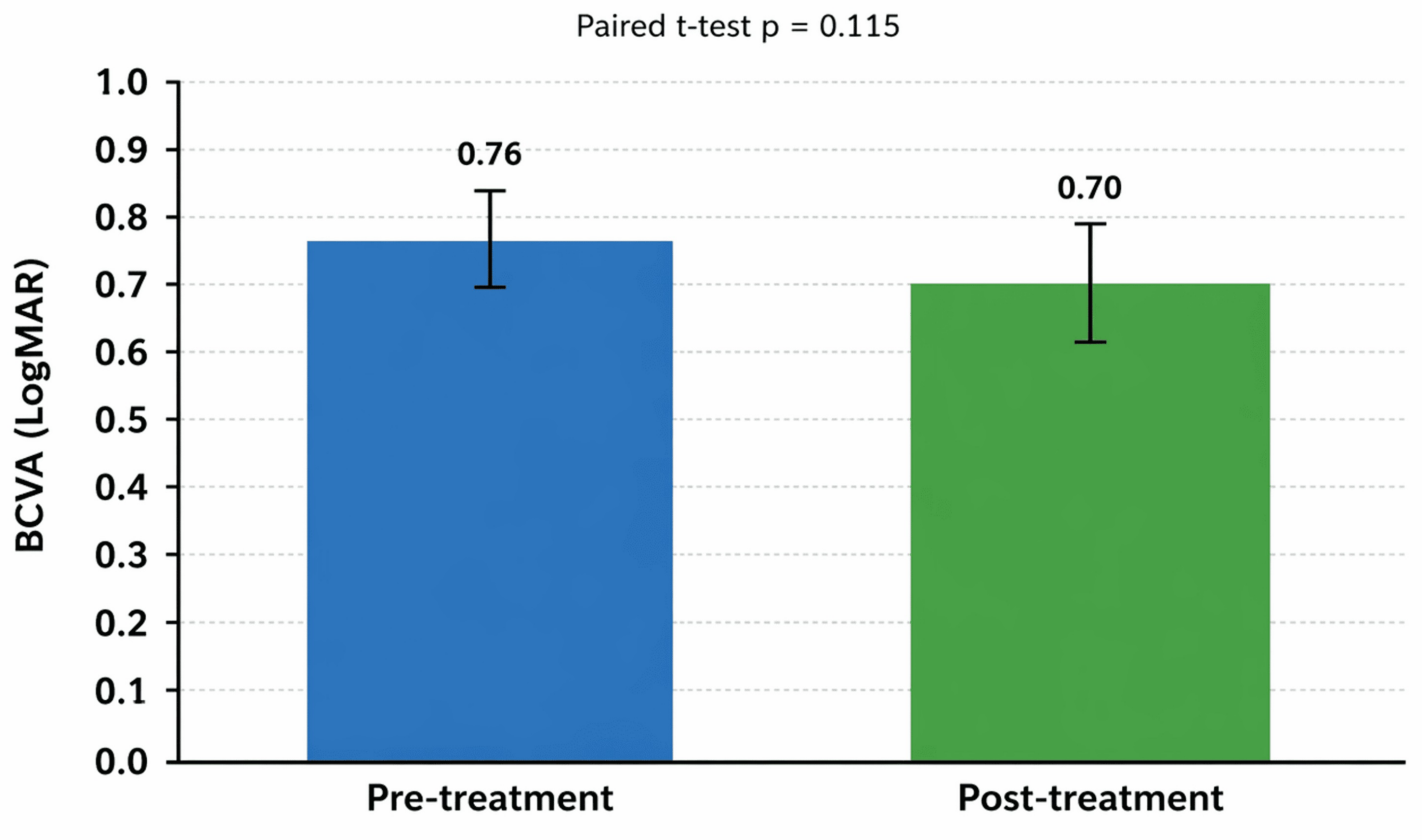
Mean BCVA before and after intravitreal adalimumab in patients with retinitis pigmentosa (n = 21). Bar graph showing the mean LogMAR BCVA at baseline and after six months of treatment. Values are mean ± standard error of the mean (SEM). A lower LogMAR value indicates better visual acuity. Visual acuity remained stable throughout follow-up, with a slight improvement from 0.76 to 0.70 LogMAR (mean Δ = −0.06). The paired t-test did not demonstrate statistical significance (p = 0.115), indicating functional preservation without evidence of treatment-related decline. Created using GraphPad Prism (GraphPad Software, San Diego, USA). BCVA: best-corrected visual acuity; LogMAR: logarithm of the minimum angle of resolution

A total of 21 patients diagnosed with retinitis pigmentosa had paired best-corrected visual acuity (BCVA) measurements, calculated as the mean LogMAR of both eyes. Mean BCVA showed a slight improvement from 0.76 at baseline to 0.70 after intravitreal adalimumab treatment (Δ mean = −0.06 LogMAR; 95% CI [−0.13, 0.02]). Normality of paired differences was acceptable (Shapiro-Wilk p = 0.099), and the paired t-test did not demonstrate a statistically significant change (p = 0.115). These findings showed no statistically significant change and are consistent with the expected range of test-retest variability in functional measurements in advanced retinitis pigmentosa (Figure 1).

Visual field outcomes 

**Figure 2:**
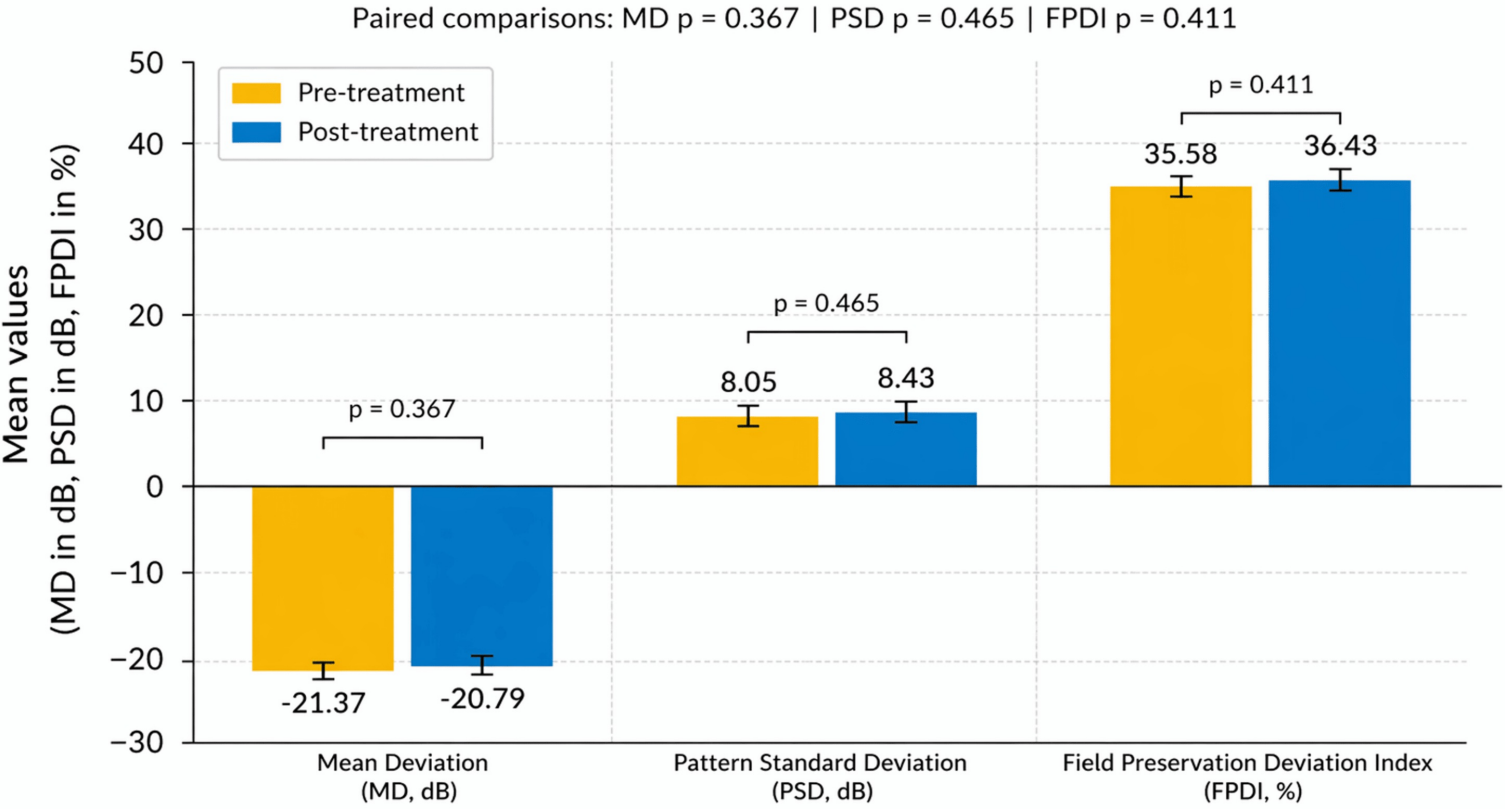
Visual field outcomes before and after intravitreal adalimumab in patients with retinitis pigmentosa. Bar graph illustrating the mean values of mean deviation (MD), pattern standard deviation (PSD), and Field Preservation Deviation Index (FPDI) at baseline and after six months of treatment. Values are mean ± standard error of the mean (SEM). For MD and PSD, higher (less negative) values indicate better outcomes. For FPDI, higher values indicate better preservation of the visual field. MD and PSD remained stable over time (MD: −21.37 to −20.79 dB, p = 0.363; PSD: 8.05 to 8.43 dB, p = 0.465), while FPDI showed a non-significant trend toward improvement (35.5% to 38.1%, p = 0.431). These findings indicate functional preservation of the visual field without evidence of deterioration following treatment. Created using GraphPad Prism (GraphPad Software, San Diego, USA).

Visual field parameters were evaluated in patients diagnosed with retinitis pigmentosa using automated perimetry. For mean deviation (MD), the mean values remained stable, changing from −21.37 ± 6.8 dB before treatment to −20.79 ± 7.1 dB after treatment (p = 0.363, paired t-test). Similarly, pattern standard deviation (PSD) showed no significant variation (8.05 ± 2.7 dB vs. 8.43 ± 2.6 dB; p = 0.465). The Field Preservation Deviation Index (FPDI) showed no statistically significant change from 35.5% to 36.4% (p = 0.411), remaining within the expected range of test-retest variability in patients with advanced retinitis pigmentosa (Figure 2). Overall, no statistically significant changes were observed across any of the three visual field parameters.

Flicker electroretinography (ERG) outcomes 

**Figure 3:**
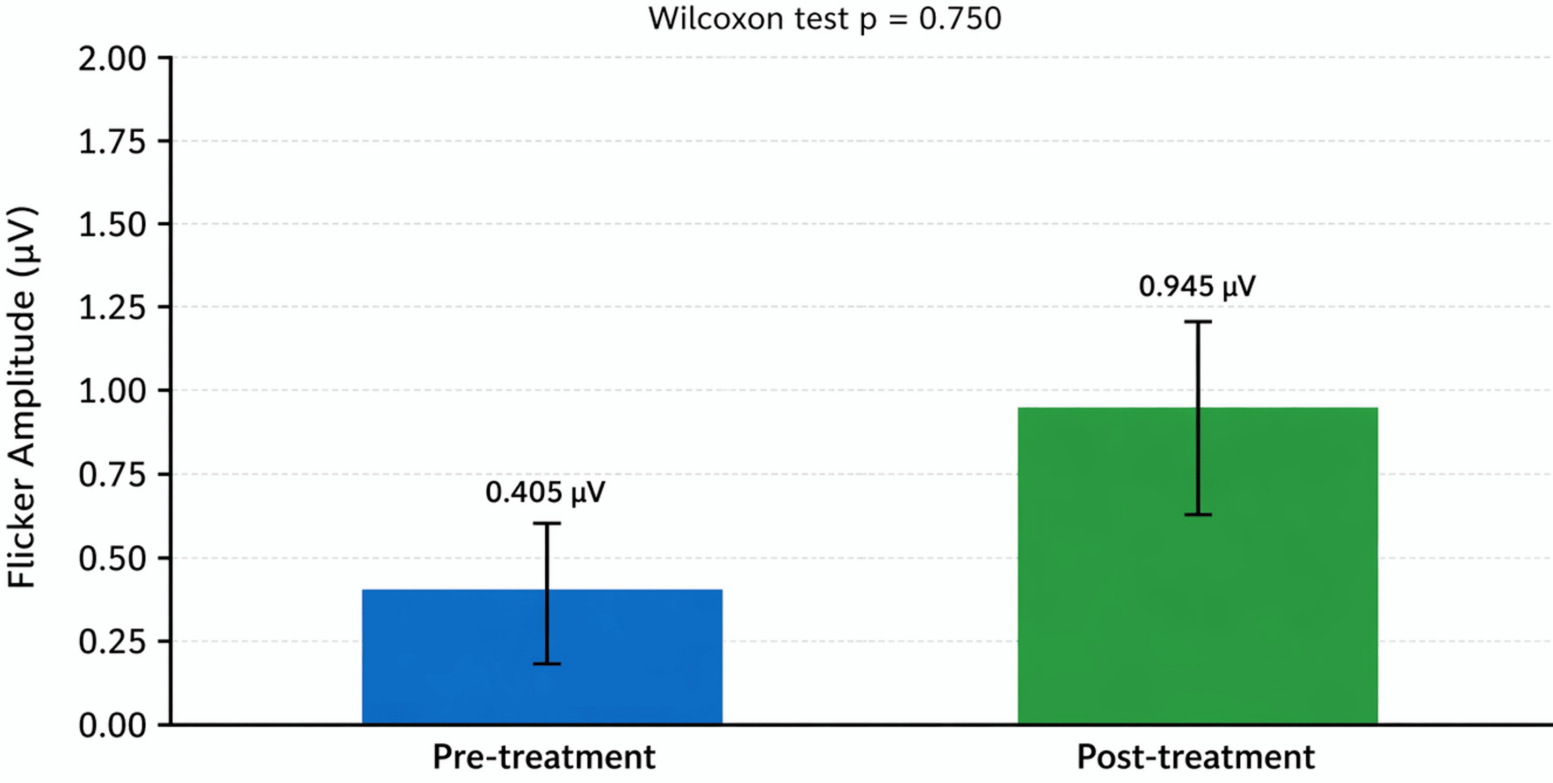
Flicker ERG (30 Hz, Phase 1) responses before and after intravitreal adalimumab in patients with retinitis pigmentosa. Bar graph showing the median 30-Hz flicker amplitude recorded at baseline and after treatment. Values are mean ± standard error of the mean (SEM). Flicker ERG responses were measurable in only five patients due to the advanced stage of retinal degeneration in this cohort. Amplitudes remained stable, increasing slightly from 0.405 µV to 0.945 µV, with no statistically significant difference (p = 0.750, Wilcoxon test). These findings indicate preservation of residual cone-mediated function without evidence of electrophysiological deterioration following intravitreal adalimumab therapy. Created using GraphPad Prism (GraphPad Software, San Diego, USA). ERG: electroretinography

Flicker ERG (30 Hz, phase 1) was available in only a small subset of patients with retinitis pigmentosa (n = 5) due to the advanced stage of disease and limited measurable responses. The median amplitude showed minimal variation, increasing slightly from 0.41 µV at baseline to 0.95 µV after treatment (median Δ = −0.06 µV; IQR (−0.38; +0.32); p = 0.75, Wilcoxon test). No consistent or statistically significant change was observed in latency, as most post-treatment responses were below the measurable threshold. Overall, the flicker ERG responses remained stable throughout follow-up (Figure 3).

Optical coherence tomography (OCT) findings

Structural evaluation with optical coherence tomography (OCT) was performed in all patients to assess potential retinal changes during follow-up. At baseline, OCT revealed marked disruption of the ellipsoid zone and reduced central macular thickness, consistent with the advanced stage of retinitis pigmentosa in this cohort. Quantitative ellipsoid zone (EZ) length analysis was not feasible in most eyes due to advanced photoreceptor loss and extensive baseline ellipsoid zone disruption. However, during the follow-up period, no additional structural changes were detected in either the ellipsoid zone integrity or the macular thickness, and no cystoid macular edema was observed. These findings showed no statistically significant change and should be interpreted within the expected range of test-retest variability in advanced retinitis pigmentosa.

## Discussion

The present prospective, single-arm pilot study was designed to evaluate the short-term ocular safety, feasibility, and exploratory signals associated with intravitreal adalimumab in patients with retinitis pigmentosa (RP). Over a six-month follow-up period, treatment was well tolerated, with no serious ocular adverse events observed. Across multimodal assessments, including best-corrected visual acuity (BCVA), automated perimetry, flicker electroretinography (ERG), and optical coherence tomography (OCT), no statistically significant changes were detected.

These findings should be interpreted within the context of an exploratory, uncontrolled pilot design. Rather than demonstrating efficacy, the results primarily support the feasibility of repeated intravitreal administration of adalimumab and provide preliminary estimates of variability and effect size that may inform the design of future clinical trials. In this regard, the present study contributes to an emerging body of translational research exploring anti-inflammatory strategies in inherited retinal degeneration [9,22].

**Figure 4:**
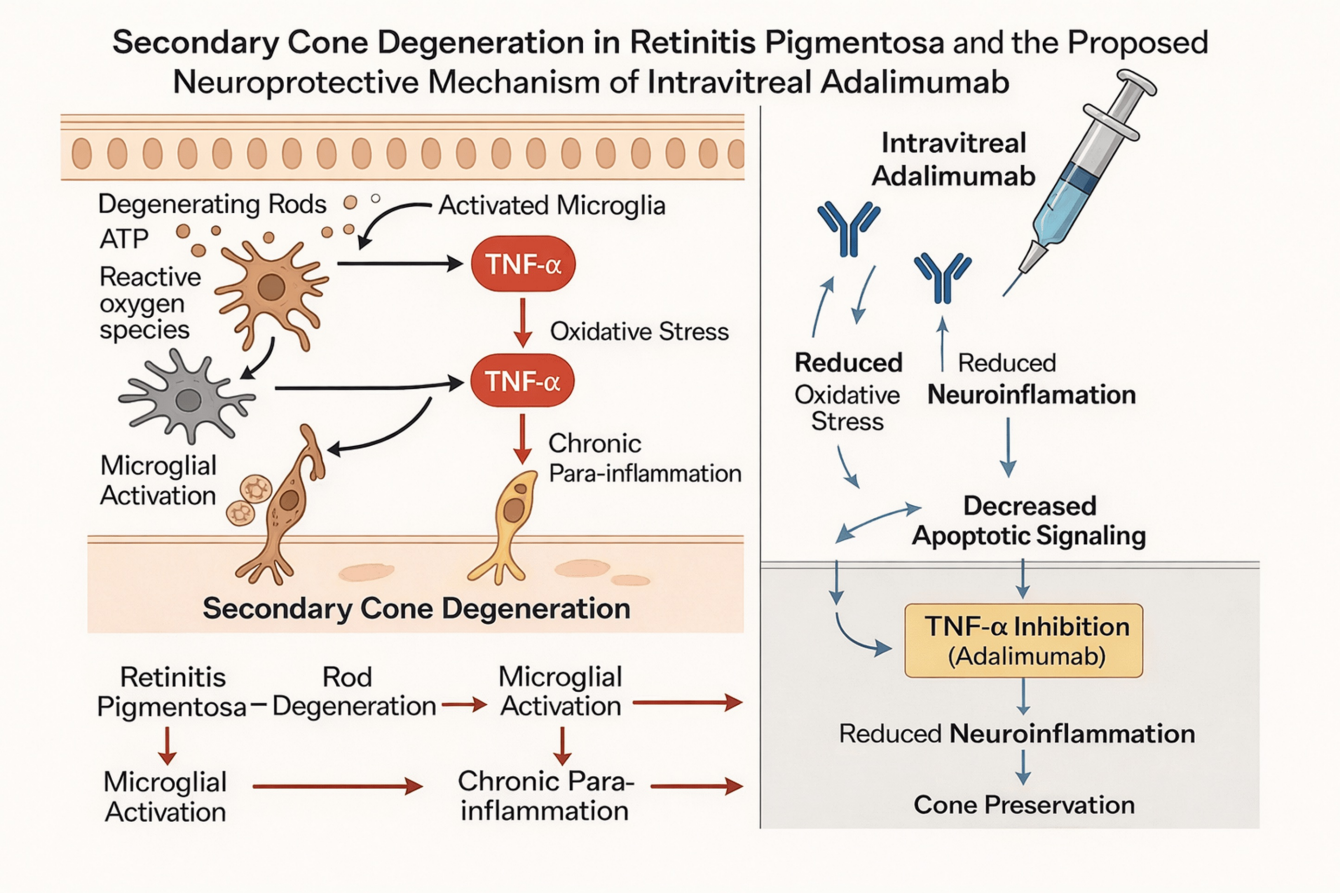
Mechanistic model of TNF-α–driven para-inflammation and secondary cone degeneration in retinitis pigmentosa, and the proposed neuroprotective effect of intravitreal adalimumab. This schematic illustrates how chronic para-inflammation contributes to secondary cone degeneration in retinitis pigmentosa (RP). Degenerating rods release ATP, reactive oxygen species, and other damage-associated signals that activate microglia and stimulate local production of TNF-α. Elevated TNF-α amplifies oxidative stress, complement activity, and chronic para-inflammatory signaling, ultimately promoting cone apoptosis and structural decline. On the right, the proposed therapeutic mechanism of intravitreal adalimumab is depicted. By neutralizing TNF-α, adalimumab is hypothesized to attenuate microglial activation, reduce oxidative and complement-mediated injury, suppress downstream neuroinflammatory pathways, and thereby help preserve remaining cones. The conceptual framework supports investigation of TNF-α blockade as a potential neuroprotective strategy in RP. Created using BioRender.com (BioRender, Toronto, Canada). No AI-powered tools or AI-assisted features within BioRender were used in the creation of these images. ATP: adenosine triphosphate; TNF-α: tumor necrosis factor alpha

A growing body of experimental and clinical evidence suggests that chronic para-inflammatory processes, including microglial activation and increased expression of pro-inflammatory cytokines such as tumor necrosis factor alpha (TNF-α), may contribute to secondary photoreceptor degeneration in RP [3,6,7,8]. This inflammatory milieu has been associated with oxidative stress, complement activation, and progressive cone loss following rod degeneration [7,9]. This biological framework provides a rationale for investigating TNF-α inhibition as a potential therapeutic approach (Figure 4).

However, it is important to emphasize that the current study was not designed to test mechanistic hypotheses or to establish a disease-modifying effect. Any interpretation linking TNF-α inhibition to functional or structural stability remains speculative and requires validation in controlled, biomarker-driven studies [24].

In this cohort of patients with advanced RP, functional parameters remained overall stable during the follow-up period. Mean BCVA showed a small, non-significant numerical improvement, while visual field indices (MD, PSD, and FPDI) did not demonstrate statistically significant changes. These observations must be interpreted cautiously. In advanced stages of RP, functional measurements are subject to substantial test-retest variability and potential floor effects, particularly over short observation intervals [9]. Therefore, the absence of a measurable decline cannot be interpreted as evidence of a treatment effect. Instead, these findings should be considered descriptive and hypothesis-generating.

Electrophysiological assessment using 30-Hz flicker ERG was limited to a small subset of patients (n = 5), reflecting the advanced disease stage and reduced detectability of responses in this population. Given the very limited sample size, ERG findings were analyzed descriptively without inferential statistical testing. No consistent pattern of change was observed over time. These results primarily support the absence of an overt electrophysiological safety signal, rather than providing evidence of functional preservation or improvement. Previous experimental and clinical studies have similarly suggested that intravitreal adalimumab does not induce retinal toxicity, supporting its ocular safety profile [11,13,14,16].

Structural OCT findings were consistent with advanced RP at baseline, including marked disruption of the ellipsoid zone and reduced macular thickness. During follow-up, no evidence of treatment-related anatomical deterioration or cystoid macular edema was observed. While these findings suggest structural stability, their interpretation is limited by the reduced sensitivity of OCT metrics in advanced disease and the relatively short duration of follow-up. As such, no conclusions regarding structural efficacy can be drawn. The absence of cystoid macular edema, a condition frequently associated with inflammatory dysregulation in RP, may indirectly support the absence of pro-inflammatory exacerbation during treatment [19].

From a clinical perspective, the present findings support the short-term ocular safety and feasibility of intravitreal adalimumab in a small cohort of patients with RP. The absence of serious ocular adverse events is particularly relevant given the off-label use of a monoclonal antibody delivered via the intravitreal route. Previous studies investigating intravitreal adalimumab in inflammatory ocular diseases have also reported acceptable safety profiles, further supporting its feasibility in selected contexts [14,16,17].

This study has several important limitations. The single-arm design without a control group precludes causal inference. The sample size was small, and the study was not powered to detect clinically meaningful differences. The use of patient-level averaged outcomes may obscure inter-eye variability and limit interpretability. Electrophysiological data were restricted by low detectability in advanced disease, further reducing sensitivity for change detection. These limitations reinforce the exploratory and hypothesis-generating nature of the findings [22]. Additional limitations should be acknowledged. The study population consisted predominantly of patients with advanced retinitis pigmentosa, which may limit sensitivity to detect functional or structural changes and restrict generalizability to earlier disease stages. Genetic heterogeneity was not systematically analyzed, and genotype-stratified effects could not be explored. The use of patient-level averaging for BCVA does not account for inter-eye correlation and may reduce sensitivity to detect asymmetric responses. Furthermore, the relatively short follow-up period of six months may be insufficient to capture meaningful changes in a slowly progressive condition such as retinitis pigmentosa. These factors further reinforce the exploratory and hypothesis-generating nature of the present findings.

Future studies should prioritize randomized controlled designs with adequate statistical power, clearly defined analytic units (eye-level or patient-level with appropriate modeling), and longer follow-up periods. The integration of objective biomarkers, including quantitative imaging metrics and molecular indicators of inflammation, may improve sensitivity for detecting treatment-related effects. Preclinical and translational studies suggest that modulation of inflammatory pathways, including TNF-α signaling, remains a promising avenue for investigation in inherited retinal degeneration [20,21,24].

In summary, this prospective pilot study provides preliminary evidence that intravitreal adalimumab can be administered safely and feasibly in patients with retinitis pigmentosa over a six-month period. The findings should be interpreted as hypothesis-generating and serve primarily to support the development of future controlled studies aimed at rigorously evaluating the safety and potential therapeutic role of TNF-α inhibition in this population.

## Conclusions

This prospective single-arm pilot study suggests that intravitreal adalimumab is feasible to administer and demonstrates a favorable short-term ocular safety profile in patients with retinitis pigmentosa. No statistically significant functional or structural changes were observed over the six-month follow-up period.

Given the uncontrolled design, small sample size, and limited statistical power, these findings should be interpreted as hypothesis-generating. The results provide preliminary data to inform the design of future controlled studies aimed at evaluating the safety and potential role of TNF-α inhibition in inherited retinal degeneration.
